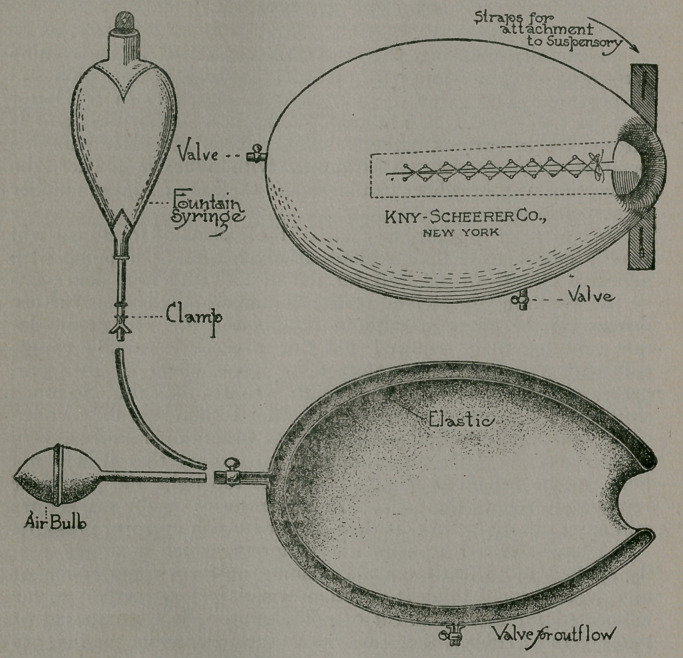# Pneumatic Scrotal Compressor for Use in Epididymitis

**Published:** 1907-12

**Authors:** Edgar Ballenger

**Affiliations:** Lecturer on Genito-Urinary Diseases, Atlanta School of Medicine, Atlanta, Ga.


### PNEUMATIC SCROTAL COMPRESSOR FOR USE IN EPIDIDYMITIS.

By Edgar Ballenger, M. D.,

LECTURER ON GENITO-URINARY DISEASES, ATLANTA SCHOOL OF MEDICINE, ATLANTA, GA.

Generally speaking, the chief factors desired in the routine treatment of epididymitis are rest, cold or heat, compression and suspension. The value of compression, especially in the latter part of the disease, is admitted by the best authorities, and is attested by the various methods that have been advocated with this purpose in view, such as strapping with adhesive plaster, the application of thin rubber bandages and the use of a large number of suspensories.

An attempt to utilize any one of these plans of treatment will readily demonstrate their serious disadvantages. Strapping with adhesive plaster works well for the first twenty-four hours until the swelling has yielded to the compression, then no further benefit can be obtained without re-applying it. The manipulation and handling required for this procedure usually causes sufficient irritation to counteract the good effect of the compression. The plaster also frequently irritates the skin. So much skill and judgment is necessary in order to bandage the swollen part with thin rubber, that the average practitioner finds it very unsatisfactory. It is either applied so loosely that it will not hold and no benefit is obtained, or it is too tight and has to be removed to relieve the pain. The suspensories on the market are still more unsatisfactory, as they do not furnish uniform pressure.

I have devised a pneumatic compress, which is simple, easily applied and effective in maintaining uniform compression of any degree, and I think it is the most satisfactory method so far advocated for the treatment of epididymitis. It is composed of two layers of rubber, the outer one is inelastic, while the inner is elastic. The space between them is to be inflated with air after the compressor has been applied and laced around the swollen part.

The pressure can be accurately regulated by gradually forcing the air in it, or allowing it to escape. As the swelling subsides, more air should be injected to maintain the desired pressure. A commendable feature is that this can be done without any manipulation to aggravate the condition. A light gauze stocking should be placed under it to absorb the perspiration. By using this method of treatment, patients can be gotten out of bed more promptly and with little danger of a relapse.

Local medication in the form of guaiacol in a 10 to 30 per cent. solution in glycerin, or tincture of iodine painted on the swollen part once or twice daily may be used in conjunction with the compression. These I have found to be the most useful local remedies.

The larger size compressors are supplied with an exit valve for drainage, and instead of using an ice bag in the beginning of the disease, a fountain syringe containing ice water (or hot water, if preferred) may be connected to furnish hydraulic pressure in the place of air. By regulating the height of the bag and the rate of outflow, both compression and cold can be adjusted to suit the comfort of the patient and the lessened swelling. It is well known that the benefit of an ice bag can be augmented by pressure to force some of the blood out of the tissues and prevent the too rapid dissipation of the cold.

The compresses are manufactured in several sizes and the smallest one that can be used with comfort should be selected and snugly laced before being inflated. They are attached by straps and held in place as an ordinary suspensory to prevent any tension on the cord. Their simplicity, with the almost universally admitted value of uniform pressure, which can be maintained and easily regulated or combined with cold or heat, make unnecessary any further exposition of the advantages to be derived from this plan of treatment.

1014—15 Century Bldg.

### DR ARCHIBALD SMITH.

Within recent years societies for sanitary and moral prophylaxis have been formed in many of the large cities and are doing a most noble work . These societies are not merely organizations for the betterment of sanitary and moral conditions as is supposed by some, but have for their specific aim and object the abatement of the great social evil. It has long been known that promiscuous relations between the sexes was productive of much evil, but the great amount of human suffering due to this cause and especially to diseases resulting therefrom, has not been even dreamed of till viewed in the light of modern medical science and is not now fully understood or appreciated even by the medical profession.

That venereal diseases alone are sufficient cause for a concerted movement against existing conditions, I think no one will deny after hearing the statement of Dr .Ballenger, so I will not go further into that phase of the question, as there are many points worthy of attention. Right here in our part of this country, one of the most important questions is miscegenation, or mixture of the races, and right here I might add that the yellow peril from across the sea is not near so serious as the one we have right at our doors.

The claim that the negro women form a great safeguard to the virtue of our Southern white women has a small element of truth in it, in so far as the greater ease with which their favors? may be procured, may save the virtue of a few white women, but even this is at too great a cost as a little thought will show.

---

* Read before the Fulton County Medical Association, Oct. 7, 1907.

Lawful marriage between the races is not allowed in the South, but illicit intercourse is winked at as the great number of mulattoes will prove. Now let us consider who and what these people are. They are just as much the flesh and blood of white fathers, as they are of their black mothers, and many of them sons and daughters of our best known men, and half brothers and sisters to some of our purest and noblest white women. These people are said to be improved negroes, but they are just as truly degenerated white men, and the improvement does not raise the black half one thousandth part as much as it lowers the white half. A white man before consorting with a negress should remember that this is not simply an act of pleasure, but is likely to bring human beings into the world which are his own flesh and blood, but are cursed with both illegitimacy and mongrelism, have a dissolute negress for a mother and are destined to be reared in surroundings where virtue is well nigh impossible and disease is rampant.

Few men would wish such a curse on the innocent children of their worst enemies, but thousands carelessly barter the birth right of their unborn children for a vile "mess of pottage" to gratify their own lust. Those who claim such great consideration for our white women, should also take into account the number of pure and innocent wives and children infected with vile and dangerous diseases by their husbands and fathers who have contracted them from filthy negresses. Also that the race question is largely brought about by such relations, especially in the matter of assault on white women, for so long as the white man does not let the black man's woman alone, it is much harder to convince th black man that he must not touch the white woman.

Illegitimacy is also a matter worthy of consideration, even though concerned with a mixture of races, for no one can deny that an illegitimate child is at a greater disadvantage in the battle of life, besides being a much greater burden on those who must support it, than one born in wedlock.

Another very serious matter of the present day is that of abortion, which is becoming frightfully common among respectable women, as well as those illegitimately pregnant. This when not scientifically done in cases where one life must be sacrificed to prevent the loss of both, causes not only the ruthless destruction of an innocent human being who is not able to lift even a feeble cry in its defense, and who, though yet unborn, is nevertheless a living human from the time of conception and entitled to consideration as such. Not only does this practice destroy the innocent by thousands, but has wrecked the health and taken the lives of multitudes of women who shirked the responsibilities of motherhood.

If these were all of the evils coming from improper relations of the sexes, it would be ample reason for taking vigorous action to suppress them, but in addition to the above time would fail us to tell of the divorces, breach of promise suits, killings under the unwritten law and kindred difficulties, all of which are attended with their untold heartaches and blighted lives.

Now the cause of such great evils being made clear, the next question is what are we going to do about it.

Let us consider what has been done in the past and what the results, both of these questions may be answered by two words; almost nothing. Of course, certain laws have been enacted and customs followed in regard to sex relations, but these are evidently inadequate. The two chief policies pursued in the matter have been a let alone policy, holding that matters of sex not be mentioned or discussed, especially among young people, for they would soon learn enough about them and should not have them brought to their attention. The other policy being that of reglemenlation, or an attempt to inspect all female prostitutes and confine those that are diseased. This method has some warm supporters and has caused much discussion, but can be dismissed here with a few words; first, because it is impossible; second, because it has been tried and found wanting; and third, because it is not only unfair, but leaves out of consideration the great majority of diseased persons, namely, the men; and lastly, because it is encouraging promiscuity, making it apparently safe and easy for the male fomicator to commit sin.

The policy of inaction has condemned itself by the fact that it has failed, as is abundantly proven by the remarks already made.

The three forces which seem to me to promise the most good are education, legislation and religion. Innocence is always desirable, but ignorance is not synonymous with it as many seem to think, and a chaste knowledge is far better than an impure ignorance. Solomen, who was noted for his experience with the fair sex and his great wisdom, sounds many notes of warning to young men and in at least seven cases denotes the young man who went astray following after "strange women" as "among the simple ones" or "a youth void of understanding." We educate the young people of both sexes in all manner of arts and sciences, except the most important; the science of life and right living; and expect them to learn that through instinct, chance acquaintances or bitter experiences. Would it not be far better to put before them scientific facts in a plain but scientific way, show them the horribleness of sin, also the insidiousness of temptation and how it may be combated; that a clean, pure and healthy mind in a healthy body is a great aid to pure living. Show the boys and young men that the purity and virtue of women is a sacred thing not to be trifled with and that those who have lost them are objects of pity as well as censure and not to be ruthlessly pushed further down the scale of humanity to make a space into which others may fall and recruit this great army. Above all, forestall and combat the pernicious and false theory that sexual intercourse is necessary to life and health of men at all times, and that virtue is an impossibility, except among weaklings, for so long as this theory prevails legislation and religion can avail little against what is inevitable.

In the matter of legislation, considerable changes are needed chief among which are raising the age of consent, so that a girl may not give or barter away her virtue before she reaches mature womanhood, and making more stringent and effective, the laws regarding abortion, so that the difficulty of detecting and punishing the perpetrator may be lessened.

In the matter of religion, the clergy should take more interest, or at least more active interest in these matters, give them more study and not be satisfied with merely alluding to them from the pulpit, as is the case in the majority of instances.

## Figures and Tables

**Figure f1:**